# Glycosylation of Zika Virus is Important in Host–Virus Interaction and Pathogenic Potential

**DOI:** 10.3390/ijms20205206

**Published:** 2019-10-21

**Authors:** Nanda Kishore Routhu, Sylvain D. Lehoux, Emily A. Rouse, Mehdi R. M. Bidokhti, Leila B. Giron, Alitzel Anzurez, St Patrick Reid, Mohamed Abdel-Mohsen, Richard D. Cummings, Siddappa N. Byrareddy

**Affiliations:** 1 Department of Pharmacology and Experimental Neuroscience, University of Nebraska Medical Center, Omaha, NE 68198, USA; nanda.kishore.routhu@emory.edu (N.K.R.); mehdi.bidokhti@unmc.edu (M.R.M.B.); 2 Beth Israel Deaconess Medical Center, National Center for Functional Glycomics, Boston, MA 02115, USA; slehoux@bidmc.harvard.edu (S.D.L.); rcummin1@bidmc.harvard.edu (R.D.C.); 3 Beth Israel Deaconess Medical Center Glycomics Core, Boston, MA 02115, USA; erouse@bidmc.harvard.edu; 4 The Wistar Institute, Philadelphia, PA 19104, USA; lgiron@wistar.org (L.B.G.); aanzurez@Wistar.org (A.A.); mmohsen@wistar.org (M.A.-M.); 5 Department of Pathology and Microbiology, University of Nebraska Medical Center, Omaha, NE 68198, USA; patrick.reid@unmc.edu; 6 Department of Genetics, Cell Biology and Anatomy, University of Nebraska Medical Center, Omaha, NE 68198, USA; 7 Department of Biochemistry and Molecular Biology, University of Nebraska Medical Center, Omaha, NE 68198, USA

**Keywords:** host–virus interactions, Zika virus, envelope (E) protein, glycoprotein, N-linked glycans, mass spectrometry, lectin array, host cell surface glycans

## Abstract

Zika virus (ZIKV) is a global public health issue due to its association with severe developmental disorders in infants and neurological disorders in adults. ZIKV uses glycosylation of its envelope (E) protein to interact with host cell receptors to facilitate entry; these interactions could also be important for designing therapeutics and vaccines. Due to a lack of proper information about Asn-linked (N-glycans) on ZIKV E, we analyzed ZIKV E of various strains derived from different cells. We found ZIKV E proteins being extensively modified with oligomannose, hybrid and complex N-glycans of a highly heterogeneous nature. Host cell surface glycans correlated strongly with the glycomic features of ZIKV E. Mechanistically, we observed that ZIKV N-glycans might play a role in viral pathogenesis, as mannose-specific C-type lectins DC-SIGN and L-SIGN mediate host cell entry of ZIKV. Our findings represent the first detailed mapping of N-glycans on ZIKV E of various strains and their functional significance.

## 1. Introduction

Zika virus (ZIKV) is mainly transmitted to humans via infected mosquitoes, though other transmission routes, such as through placenta and sexual intercourse, can also occur [1,2]. Recently, the considerable increase in ZIKV infection rates has raised urgent global urgent concerns, especially after the outbreaks in Yap Islands (2007) [3,4], French Polynesia (2013) [5,6], Easter Island (2014) [7], and Brazil (2015) [8,9]. These ZIKV outbreaks were associated with a sharp increase in cases of Guillain Barre Syndrome (GBS), an autoimmune disease featured by weakening and paralysis of the limbs and face. In 2015, Zika virus infection spread out to South and Central America, infecting thousands of people in Brazil and Colombia, where it was associated with an increase in GBS rates, as well as a significant increase in severe fetal abnormalities, including spontaneous abortion, stillbirth, hydrocephaly, microcephaly, hydranencephaly/hydrops fetalis, and placental insufficiency [10,11,12,13,14]. ZIKV is a small, enveloped positive-strand RNA virus [15]. The genomic RNA of this mosquito-borne flavivirus contains a single open reading frame (ORF) that encodes a polyprotein [16,17]. This polyprotein undergoes co- and post-translational processing to produce three structural proteins, which are capsid (C), pre-membrane (prM), and envelope (E), in addition to seven non-structural proteins (NS1, NS2A, NS2B, NS3, NS4A, NS4B, and NS5) [17,18]. Of the three structural proteins, E is the major surface glycoprotein, containing three domains (I, II, III) and two transmembrane helices [17,18,19]. The majority of flavivirus E proteins upon post-translational modification are modified by N-glycans at amino acid 153/154 within a highly conserved glycosylation motif of N-X-T/S at positions 154–156 (where X is any amino acid except proline) [16]. This is a primary target of neutralizing antibodies and is required for virus entry [20]. 

Previously, it was demonstrated that the N-linked glycosylation sites on the E protein of flaviviruses are highly conserved, playing a vital role in both infectivity and assembly [16,21,22,23]. Loss of N-glycosylation motif on domain I of E protein (N154) results in impaired expression and secretion of E ectodomain from mammalian cells [24,25]. Furthermore, studies in mice have shown that ZIKVs lacking the N-glycans of the ZIKV E were severely compromised for their ability to cause mortality and neuroinvasion, suggesting a vital role for these glycans of the E protein in ZIKV pathogenesis [22,26]. In this study, we utilized mass spectrometry (MS) and a lectin microarray to analyze the structures and composition of glycans on ZIKV E protein from different strains and on the surface of the virus-producing cells, as well as to explore mosquito–human mode of ZIKV transmission and its association to neurological disorders. Importantly, our functional studies show that these N-glycans of the ZIKV envelope glycoprotein are important in viral infection of cells. Our findings might have implications in understanding the roles for these glycan patterns on ZIKV E in virus–host interaction and pathogenic potential. 

## 2. Results

### 2.1. MS Analysis of ZIKV E Derived from Different Cell Lines Showed Various Profiles of N-glycan Pattern

**Figure 1:**
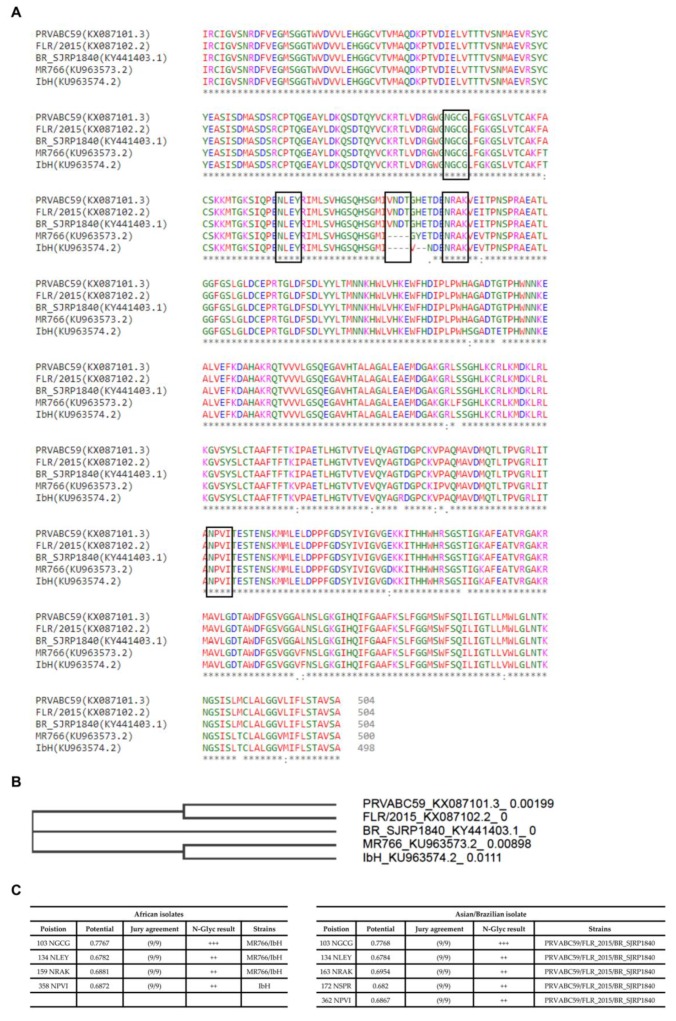
An overview of both glycoprotein E sequence variations between various ZIKV strains, and site-specific N-glycan distribution. (**A**) Multiple sequence alignment of glycoprotein E sequences of various ZIKV strains. The protein sequences of ZIKV strains MR766 (KU963573.2), PRVABC59 (KX087101.3), FLR (KX087102.2), IbH (KU963574.2), and BR_SJRP1840 (KY441403.1) were aligned using Clustal omega. Positions of the potential N-linked glycosylation sites are indicated in the boxed areas. (**B**) A phylogenetic tree (neighbour-joining tree without distance corrections) constructed based on the multiple sequence alignments of glycoprotein E from different ZIKV strains. Consensus sequences for N-linked glycosylation are highlighted in the square box. (**C**) The NetNglyc server was used to examine the sequence context of N-glycosylation sites (Asn-Xaa-Ser/Thr) in E proteins, and we depict the N-glycosylation sites at the individual ZIKV E protein sequence sites. +++ N-glycosylation sites very likely to be glycosylated.

In order to understand ZIKV E protein-associated N-glycans, we initially looked for the characteristic features of the envelope protein(s). After generating alignments of the available ZIKV strains of Asian and African origin on the sequences retrieved from GenBank, the sequences were checked by phylogenetic tree (neighbour-joining tree without distance corrections), followed by NetNglyc analysis. This analysis was performed to better understand the variations in sequences and number of N-Glycosylation sites (Asn-Xaa-Ser/Thr, where X is any amino acid except proline) between Asian strains (PRVABC59 and FLR isolates), African strains (MR766 and IbH isolates), and a Brazilian isolate from 2016 (SJRP) (Figure 1). Taken together, these analyses indicate that the number and conservation of glycosylation sites vary across different strains. Asian strains (PRVABC59 and FLR isolates) and the Brazilian isolate (BR_SJRP1840) contain five potential N-linked glycosylation sites, whereas the African strain MR766 isolate contains four and IbH isolate contains three potential N-linked glycosylation sites. However, phylogenetic tree analysis clearly indicates that the Brazilian isolate (BR_SJRP1840) clusters distinctly from Asian and African strains, denoting differences in the envelope proteins sequences.

**Figure 2:**
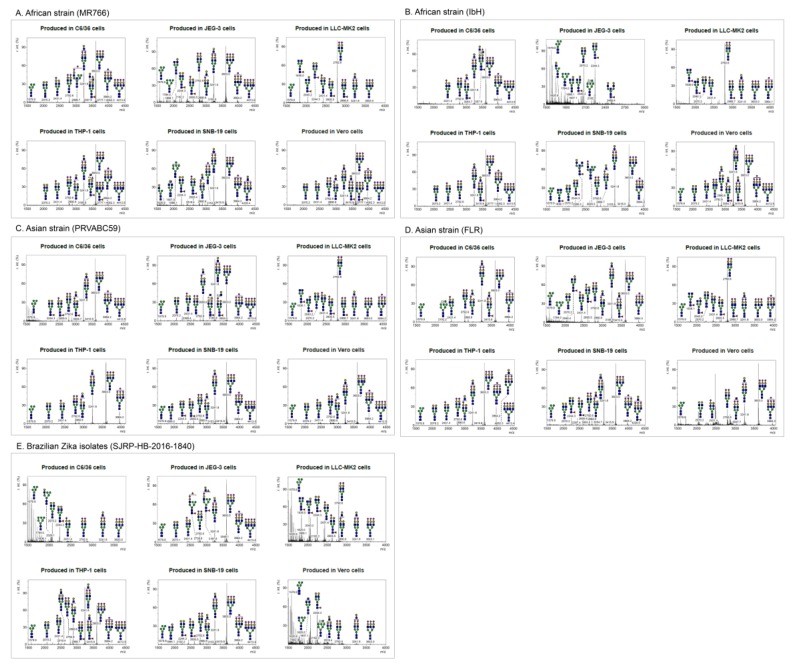
MALDI-TOF MS spectra of N-glycans on glycoprotein E of purified ZIKV. N-linked glycans were prepared from envelope protein(s) of Zika viruses (**A**) MR766, (**B**) PRVABC59, (**C**) FLR, (**D**) IbH, and (**E**) SJRP were produced from various cell types (C6/36, LLC-MK2, Vero, THP-1, SNB-19, and JEG-3 cells) and identified by MALDI-TOF mass spectrometry. The virions were purified using sucrose cushion ultracentrifugation and resolved on SDS-PAGE (see Figure S1). The presence of glycoprotein E was confirmed by Western blotting. The corresponding glycoprotein E band from mass spectrometry compatible Coomassie stained gel was excised and subjected to N-glycan release. The N-linked glycans were identified using MALDI-TOF mass spectrometry. *X* axis is mass to charge ratio (*m/z*) and Y axis represents signal intensity of the ions. Schematic representation of the N-linked glycans (high-mannose, hybrid and complex) and the corresponding molecular ions that were predicted to be identified in this study. Yellow circle, galactose; blue square, N-acetylglucosamine; green circle, mannose; red triangle, fucose; purple diamond, sialic acid.

**Figure 3:**
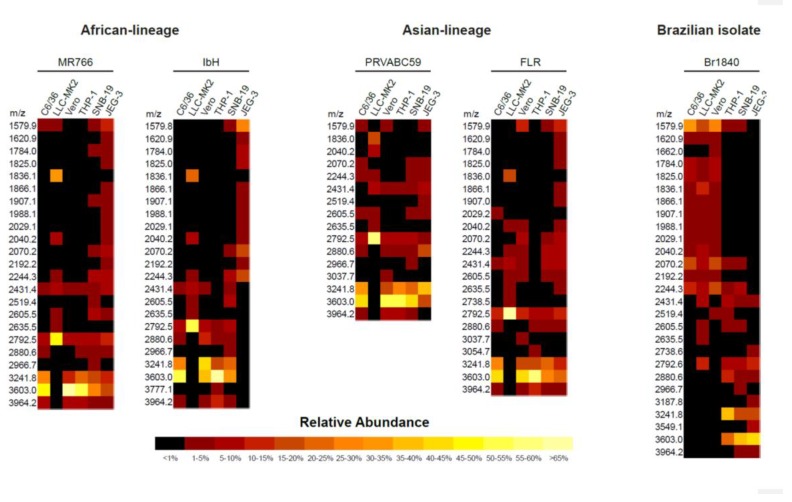
Heatmap presentation of differences in abundance of glycoprotein (E) N-linked glycans identified using MALDI-TOF MS. The heterogeneity and the abundance of glycoprotein (E)-associated N-linked glycans detected using MALDI-TOF MS between the Zika viruses produced from various cell types. The N-glycans identified through this method are represented in heatmap format.

Following the production and purification of the different ZIKV strains from various cell lines, the matured virions were pelleted from culture supernatants. The lysed cells and debris were removed by using a brief centrifugation followed by filtering through a 0.45 µm syringe filter to remove unprocessed viruses. The culture supernatants were further purified, and the pellets were lysed and resolved in 12% bis-tris protein gels and stained with Coomassie Blue (Figure S1). The bands corresponding to the ZIKV E were excised for further identification of E protein-associated N-linked glycan structures using mass spectrometry. The molecular ions in MALDI spectra of envelope protein (E) obtained from purified mature ZIKV virions produced from cell lines of different origin and the predicted glycan structures with the corresponding sizes are shown in Figure 2 (see also Table S1 in the Supplementary Material). The relative abundances of these N-glycans are presented as a heatmap in Figure 3. Although distinct N-glycosylation patterns emerged, the two ZIKV E proteins from African lineage strains, MR766 and IbH, displayed similar N-glycan profiles across the six cell lines that were tested (Figure 2A,B). We observed differential glycosylation patterns between viruses produced from different cell lines. When produced in C6/36, Vero, THP-1, and SNB19 cells, these N-glycan profiles were heavily dominated by sialylated glycans. For these four cell lines, bi- and tri-sialylated tri-antennary N-glycans (3241.8 *m/z* and 3603.0 *m/z*) were the most abundant found on ZIKV E, followed by bi-sialylated bi-antennary (2792.5 *m/z*) and tetra-sialylated tri-antennary (3964.2 *m/z*) N-glycans (Figure 2A,B). ZIKV E produced in SNB19 cells was also found to be decorated with Man5 (1579.9 *m/z*), agalactosylated bisected (1907.1 *m/z*), core-fucosylated non-sialylated bi-antennary (2244.3 *m/z*), and core-fucosylated mono-sialylated bi-antennary (2605.5 *m/z*) N-glycans (Figure 2A,B). When expressed in the LLC-MK2 and JEG-9 cell lines, the N-glycan profiles of MR766 and IbH strains of E were markedly different. When produced in LLC-MK2 cells, bi-sialylated bi-antennary (2792.5 *m/z*) and core-fucosylated agalactosylated (1836.0 *m/z*) N-glycans were by far the two most abundant glycan species (Figure 2A,B). More complex N-glycans, with three or more sialic acids and additional lactosamine repeats, were not identified in ZIKV E expressed in LLC-MK2 cells. Finally, the ZIKV E produced in JEG-3 cells revealed a different N-glycosylation pattern between MR766 and IbH strains. While the di- and tri-sialylated N-glycans (2792.5, 3241.8, and 3603.0 *m/z*) were the most abundant for the MR766 ZIKV E produced in JEG-3, the variety of N-glycans identified in JEG-3 cells was greater, notably including Man_5_GlcNAc_2_ (1579.9 *m/z*), Man_6_GlcNAc_2_ (1784.0 *m/z*), Man_7_GlcNAc_2_ (1988.1 *m/z*), Man_8_GlcNAc_2_ (2192.2 *m/z*), asialo bi-antennary (2070.2 *m/z*), and core-fucosylated asialo-bi-antennary (2244.3 *m/z*) N-glycans (Figure 2A). In contrast, the IbH strain produced in the JEG-3 cells displayed a more restricted N-glycan profile, with the notable absence of sialylated N-glycans and with the core-fucosylated asialo-bi-antennary (2244.3 *m/z*) N-glycan being the most complex N-glycan identified (Figure 2B).

Similar to African lineage ZIKV, the N-glycosylation profiles from the Asian lineage viruses PRVABC59 and FLR varied across cell lines, yet were nearly identical within any given cell line (Figure 2C,D). Additionally, the E protein of PRVABC59 and FLR strains expressed in C6/36, Vero, THP-1, and SNB19 cell lines was primarily decorated with tri-sialylated tri-antennary N-glycans (3603.0 *m/z*) and—to a lesser abundance—di-sialylated bi- and tri-antennary (2792.5 and 3241.8 *m/z*) and tetra-sialylated tri-antennary (3964.2 *m/z*) N-glycans (Figure 2C,D). Minor N-glycans, including Man_5_GlcNAc_2_ (1579.9 *m/z*), monosialylated bi-antennary (2431.4 *m/z*), core-fucosylated mono-galactosylated bi-antennary (2040.2 *m/z*), core-fucosylated asialo bi-antennary (2244.3 *m/z*), and core-fucosylated mono-sialylated bi-antennary (2605.5 *m/z*) N-glycans, were also identified (Figure 2C,D). Similar to the African lineage, the E proteins of Asian lineage revealed a more restricted N-glycan profile when expressed in LLC-MK2 cells. The di-sialylated bi-antennary (2792.5 *m/z*) N-glycan was identified as the most abundant N-glycan, with very low to no expression of more complex N-glycans. The core-fucosylated agalactosylated (1836.0 *m/z*) and mono-sialylated bi-antennary (2431.4 *m/z*) were, respectively, the second and third most relatively abundant N-glycans reported (Figure 2C,D). 

Lastly, the N-glycan profiles of the PRVABC59 and FLR strains of E expressed in JEG-3 cells showed a lower abundance of highly sialylated N-glycans such as the tri- and tetra-sialylated tri-antennary N-glycans (3603.0 and 3964.2 *m/z*) compared to the virus produced in the C6/36, Vero, THP-1, and SNB19 cells. By contrast, the E of the PRVABC59 strain showed an increased abundance of both mono-sialylated N-glycans (2431.4 and 2880.6 *m/z*) and non-sialylated N-glycans (2040.2, 2070.2, and 2244.3 *m/z*). Unlike the African and Asian lineage strains, the N-glycans found on the E protein of the Brazilian isolate SJRP were almost exclusively non-sialylated when produced in C6/36 and Vero cells. In both of these cell lines, the major N-glycans found were Man_5_GlcNAc_2_ (1579.9 *m/z*), asialo-bi-antennary (2070.2 *m/z*), and core-fucosylated asialo-bi-antennary (2244.3 *m/z*) N-glycans (Figure 2E). When produced in LLC-MK2 cells, however, the N-glycan profile of the E protein from the SJRP strain was comparable to the profiles found for the four other strains, with the di-sialylated bi-antennary (2792.6 *m/z*) and the core-fucosylated agalactosylated (1836.1 *m/z*) N-glycans being the most abundant. Contrasting the other E proteins produced in C6/36, LLC-MK2, and Vero cells, Man5 (1579.9 *m/z*) was found to be the most abundant N-glycan on the SJRP strain. However, the N-glycan profiles generated from the E protein of SJRP strain expressed in THP-1, SNB19, and JEG-3 cells revealed a high relative abundance of di-sialylated bi-antennary (2792.5 *m/z*), tri-antennary (3241.8 *m/z*), and tri-sialylated tri-antennary (3603.0 *m/z*), with little to no expression of less complex non-sialylated N-glycans such as Man_5_GlcNAc_2_ (Figure 2E). 

The major proportion (>5%) of N-glycan spectra at 3603.0, 3241.8, 2792.6, 3964.2, 1836.0, and 2040.2 mz were identified on ZIKV E produced in these cell lines. These heterogeneous N-glycan structures of the studied ZIKV E include high (oligo)-mannose; these and other N-glycans compositionally are predicted to include the residues N-acetylgalactosamine (GalNAc), N-acetylglucosamine (GlcNAc), N-acetylneuraminic acid (NeuAc), galactose (Gal), glucose (Glc), fucose (Fuc), and sialic acid. In addition, we found many other glycan forms with high heterogeneity but smaller proportions (<5%); these were not further characterized. These data are indicative of high heterogeneity of structure and composition of N-glycans on ZIKV E proteins (Figure 2 and Figure 3; see also Table S1 in the Supplementary Material).

### 2.2. Cell surface Glycosylation Strongly Correlates with Glycomic Features of ZIKV E Protein

**Figure 4:**
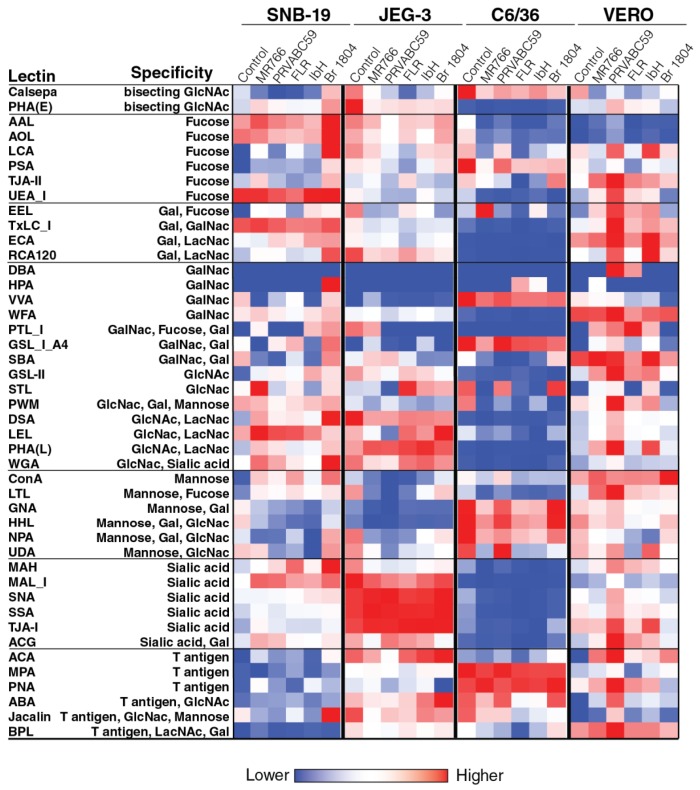
Glycomic profiling of Zika virus-infected and uninfected cell membrane using lectin microarray. Lectin microarray (45 structures) was used to analyze the glycans associated to cell surface proteins of Zika virus-infected and uninfected cells. The different cell types (C6/36 cells, Vero cells, SNB-19 cells and JEG-3 cells) were mock-infected and infected with distantly related Zika viruses (MR766, PRVABC59, FLR, IbH, and SJRP). The cell surface proteins were extracted using a membrane protein extraction kit, and labeled with Cy3 dye and hybridized to a lectin microarray. The resulting lectin chip was scanned for fluorescence intensity on each lectin-coated spot using an evanescent-field fluorescence scanner. The data were normalized using the global normalization method. The heatmap format was used to present the glycan profiles identified using this method.

In order to identify the general patterns of endogenous glycans in ZIKV-producing cell surface endogenous glycoproteins, a 45 lectin microarray was used, and the details of microarray and their names and glycan binding specificities employed in this study are shown in Table S3. This approach relies on specificities of immobilized lectins that recognize specific glycan features on cell surface glycoproteins, resulting in their binding to the array. These data revealed that different cell lines harbor differential cell surface glycosylation (Figure 4). For example, JEG-3 cells exhibit higher levels of sialic acid (measured as binding to MALI, SNA, SSA, TJAI, and ACG lectins) and GlcNac (measured as binding to DSA, LEL, PHAL, and WGA lectins) when compared to other cell lines, especially C6/36 cells. On the other hand, C6/36 cells exhibit higher levels of fucose mannose (measured as binding to ConA, GNA, and HHL lectins) and T-antigen (measured as binding to MPA, PNA, and ABA lectins) when compared to other cell lines, especially JEG-3 cells. The differential levels of glycosylation of these cells might have an impact on the glycosylation of the virus produced in these cells. 

**Figure 5:**
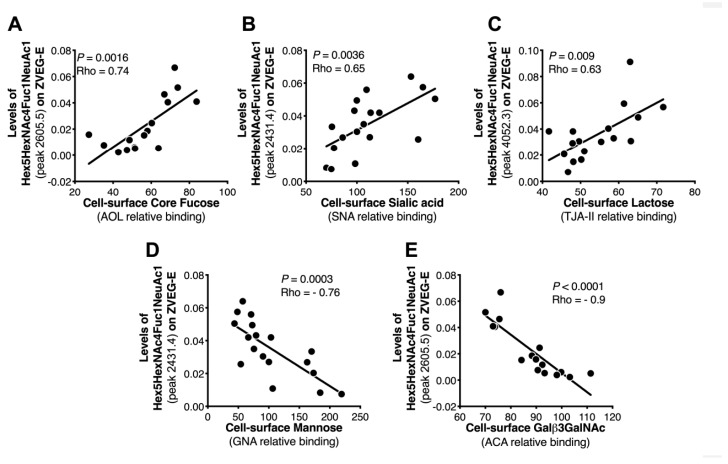
Correlation analysis between MALDI-TOF MS-measured glycans and lectin microarray-measured glycans. Correlation analysis was performed to identify the strength of relationships between glycan profiles identified by MALDI-TOF mass spectrometry and by lectin microarray. This analysis confirms a linear correlation between cell surface glycans identified by lectin microarray, i.e., (**A**) fucose, (**B**) sialic acid, (**C**) lactose, (**D**) mannose, and (**E**) GlcNAc and the glycomic features of Zika virus protein E observed by MALDI-TOF mass spectrometry.

In general, the expression of several cell surface glycan structures correlated with viral N-glycan features (Table S2). Of interest, and as shown in Figure 5, strong correlation was observed between the presence of cell surface Fuc (binding to AOL lectin, which binds to both core and branched fucose), sialic acid (binding to SNA lectin, which binds to α2,6 sialic acid), lactose (binding to TJAII lectin, which binds to both branched fucose and lactose), mannose (binding to GNA lectin), GlcNAc (binding to ACA lectin), and the N-glycan features of E protein. These data support the concept that the glycosylation machinery of the virus-producing cells may impact the glycosylation of the virus, which maybe implicates viral pathogenesis. There was no statistically significant difference with other proteins analyzed.

### 2.3. C-Type Lectins DC-SIGN and L-SIGN Play a Functional Important Role in ZIKV Infection

The C-type lectins such as DC-SIGN (dendritic cell-specific ICAM-grabbing non-integrin, where ICAM is an intercellular adhesion molecule) and L-SIGN (where L is liver or lymph node) have considerable binding affinity to viral mannose-rich glycans [27,28]. DC-SIGN is expressed on various cell types such as monocytes and dendritic cells (DCs), and L-SIGN is expressed on endothelial cells of liver and lymph nodes (L) and recognizes carbohydrate structures present on viral glycoproteins [28]. Thus, these lectins function as attachment factors for several enveloped viruses, including human immunodeficiency virus-1 (HIV-1), Ebola (EBOV), Japanese encephalitis virus (JEV), and Dengue virus (DENV) [29,30]. 

**Figure 6:**
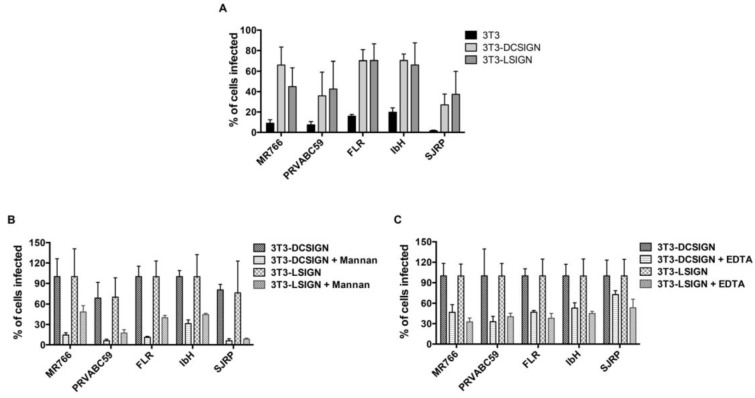
The functional importance of N-linked glycans on Zika virion infectivity. (**A**) DC-SIGN/L-SIGN-mediated enhancement of Zika virus infection. 3T3, 3T3-DC-SIGN, and 3T3-L-SIGN cells were infected with ZIKV at MOI of 1, and the expression of viral E glycoprotein was detected 24 h later using an immunofluorescence assay. (**B**) DC-SIGN/L-SIGN-mediated enhancement of Zika virus infection is inhibited by Mannan. 3T3-DC-SIGN or 3T3-L-SIGN cell lines were incubated with Mannan (at 200 μg/mL) inhibitors for 30 min at 37 °C and then infected with ZIKV at MOI of 1. The same concentration was maintained constant throughout duration of infection. (**C**) DC-SIGN/L-SIGN-mediated Zika virus infection is reduced by EDTA. 3T3-DC-SIGN or 3T3-L-SIGN cell lines were pre-incubated with EDTA (at 0.5 mM) for 30 min at 37 °C and then infected with ZIKV at MOI of 1. The treatment was continued for 2 h. After 2 h, the virus and the EDTA-containing medium were removed and the infection was continued with fresh medium. After 24 h, the infected cells were detected using viral E protein antibody and the percent of infected cells was assessed by an immunofluorescence assay using Operetta High-Content Imaging System. Error bars indicate standard deviations calculated from four replicate wells in a 96-well plate.

Given that ZIKV envelope-associated N-glycan structures are enriched with mannose, we examined the potential interactions of ZIKV E glycans with host lectins (DC-SIGN and L-SIGN), and their impact on ZIKV entry and infection. We employed 3T3/NIH cells (which were originally established from the mouse embryonic fibroblast cells) expressing DC-SIGN (DC-SIGN/3T3 cells) and L-SIGN (L-SIGN/3T3 cells) receptors, along with only 3T3/NIH cells as a control. Cells were infected with ZIKV at a MOI of 1, and the percent infection was determined after 24 h through the use of an immunofluorescence analysis and a high-throughput Operetta imager. As presented in Figure 6, the percentage (%) of infected cells increased in DC-SIGN- and L-SIGN-expressing cells compared to control 3T3/NIH cells, indicating that DC-SIGN- and L-SIGN-expressing 3T3 cells are susceptible to ZIKV infection (Figure 6A). To further confirm the role of high-mannose-type glycans, we performed experiments in the presence of inhibitor/blocker mannan and EDTA. EDTA blocks DC-SIGN by extracting the bound calcium, while Mannan competes with insect-derived high-mannose-type glycans and both inactivate DC-SIGN [31]. Both mannan and EDTA inhibited ZIKV replication efficiently (Figure 6B,C). These results suggest that ZIKV infection efficiency is dependent upon high-mannose-type glycans, and the entry is mediated through DC-SIGN/L-SIGN cell surface expression. Taken together, DC-SIGN/L-SIGN receptors are essential for optimal entry and infection of various strains of ZIKV.

## 3. Discussion

A comprehensive understanding of glycosylation pattern of the viral antigenic proteins is key towards therapeutic discovery and vaccine development. It is equally important to understand the effect of glycosylation on the biological properties and pathogenesis of the virus, which can be used for designing glycomimetic compounds as potential antiviral agents [32]. In this study, we used MS to characterize the E protein N-glycans of ZIKV derived from insect cells and mammalian cells. We showed for the first time that ZIKV E glycoproteins, which are required for host cell receptor binding, are differentially glycosylated when produced from different cell lines. The N-glycosylation pattern on ZIKV E is thought to be involved in viral infectivity, morphogenesis, protein folding, virus entry, and tissue tropism, among other functions [24,25]. This has also been described for other viruses, including influenza virus [33], HIV-1, West Nile Virus, JEV, hepatitis C virus (HCV), Nipah virus, EBOV, and sever acute respiratory syndrome (SARS) coronavirus [34]. 

In the current study, Asian strains (including strains circulating in Southeast Asia, and South and Central America) of ZIKV, but not African strains, contain the N154 glycosylation site of the E protein (Figure 1), which has been reported previously [35]. Contrasting the four African and Asian lineage viruses, the Brazilian ZIKV isolate SJRP revealed only partial similarities in N-glycosylation. It is believed that the N-linked oligosaccharides in insects cell lines have terminal mannose residues, unlike the N-linked oligosaccharides processed in mammalian cells, because mammalian cells have enzymes that can further process to produce complex N-linked oligosaccharides with terminal sialic acid, glucose, galactose, and other sugars, but, not terminal residue as mannose [36,37]. As DC-SIGN preferentially binds to terminal mannose residues [27,28], one would predict that viruses grown in insect cells would bind to this receptor and infect DCs better than viruses grown in mammalian cells. Despite those differences in N-linked glycan structures, it has been previously shown DENVs derived from both cell lines can efficiently infect DCs because of the presence of unprocessed, high-mannose glycans on the E protein [38,39,40,41]. In our study, SJRP was decorated only with non-sialylated N-glycans when generated in both C6/36 and Vero cell lines; however, mostly with sialylated N-glycans in THP-1, SNB-19, and JEG-3 cells (Figure 2, Figure 3 and Figure 4), suggesting that decoration of ZIKV E with sialylated N-glycans may correlate with diverse tissue tropism of the virus. Taken together, these findings lead us to assume that the “N-glycotype” of the recently emergent Asian strains would offer benefit of viral fitness, and would enhance the new neuropathogenic potential of ZIKV infection and also their increased infectivity in mosquitoes. 

The results of our study reveal high heterogeneity in structure and composition of the N-glycans linked to ZIKV E produced by both insect and various mammalian cells, including monocytes, placental, and neural cell types. The heterogeneity of N-glycan has also been linked to DENV E protein in terms of structure and composition [23]. The N-glycan on DENV E protein that is produced by mammalian cells is a mixture of high-mannose glycan and complex glycan [23,38]. Mosquito cells, by contrast, provide a mix of high-mannose glycan and paucimannose glycan [38]. It has been shown that ZIKV replication in macaques also restores the missing N-glycosylation site (150 loop of the E protein) in MR766 strain [42], suggesting that the passage history (host animal and cell line types) affects the E protein glycosylation pattern of ZIKV strains. Taken together, our study, for the first time, reveals glycan structures linked to ZIKV E, analyzed by using MS and lectin microarray, are varied in structure and composition depending on ZIKV strains and host cell types. 

Analyses of the N-glycans on ZIKVs grown in insect and various mammalian cells revealed a high degree of heterogeneity. The variation in N-glycans found on ZIKV E ranged from high- (or oligo-) mannose, notably Man_5_GlcNAc_2_, to highly sialylated (up to four sialic acids) complex-type N-glycans, and with very few hybrid types. However, both African and Asian lineage strains revealed very similar N-glycosylation patterns of ZIKV E. Di- and tri-sialylated bi- and tri-antennary were the most relatively abundant N-glycans found on ZIKV E when viruses were produced in C6/36 insect cells and LLC-MK2, Vero, THP-1, SNB19, and JEG-3 mammalian cells. High-mannose, asialo-, and agalacto N-glycans were also identified, though in lesser fraction overall relative abundance. The presence of high-mannose and complex-type N-glycans on mammalian cell-derived virus is not only limited to ZIKV, as has been reported earlier for DENV serotypes [38,43], but is the most abundant type of N-glycan found on HIV gp120 glycoprotein [44]. On the other hand, any ZIKV strain when produced in various cell lines showed different N-glycan patterns. Indeed, from a cell line perspective, LLC-MK2 cells produced the same unique N-glycosylation pattern for all five ZIKV E, which were mostly decorated with bi-antennary di-sialylated and core-fucosylated agalactosylated N-glycans (Figure 2, Figure 3 and Figure 4; also see Table S1 in the Supplementary Material). Collectively, these findings indicate that not only virus characteristics, e.g., strain or lineage, but also host cell physiology has an impact on the glycosylation pattern of ZIKV E, which may affect viral pathogenesis of ZIKV strains. 

The C6/36, Vero, THP-1, and SNB-19 cells produced ZIKV E N-glycosylation profiles with highly sialylated N-glycans on the E protein of all ZIKV strains, except for MR766 in JEG-3 cell and SJRP in C6/36 and Vero cells, where the N-glycosylation profile was mostly restricted to low molecular weight non-sialylated N-glycans. These data suggest that the different contexts of specific host cells could significantly affect the N-glycosylation profile of the ZIKV E of different ZIKV strains. The cell culture media, culture conditions, and sequence of a protein may also significantly influence the quality and relative abundance of N-glycans observed on the cell-produced glycoproteins [45,46]. The results of our study also revealed a high heterogeneity in the N-glycans linked to ZIKV E among different ZIKV strains that are produced by the same host cell. For example, Vero cells produced ZIKV E linked to various N-glycans among ZIKV strains, including FRL, IbH, PRVABC59, and MR766. Unlike other flaviviruses, ZIKV E contains a conserved sequence of ~10 amino acids (150 loop of the E protein) that surround the N154 glycosylation site. This conserved sequence may affect the glycosylation process, carbohydrate moiety, and receptor binding site of the ZIKV to host cells [18]. The number and location of glycosylation motifs in the E vary considerably, both between and within flaviviruses [47], altering the pathogenicity of these viruses and the severity of their diseases [22]. The African lineage of ZIKV isolates lack the N-glycosylation site (150 loop of the E protein), while ZIKV isolates from recent outbreaks in South and Central American regions, that originated from the epidemy of Asian lineage ZIKV PF-2013 in French Polynesia in 2013, contain the glycosylation site within the viral envelope [22]. Indeed, in a previous study, the recombinant ZIKV strains that lack the glycosylation site were unable to induce mortality in a mouse model [22]. However, recent studies have revealed that residues surrounding the ZIKV E protein glycan regulate virus antigenicity, irrespective of the presence of a glycan [48]. The glycosylation of ZIKV E protein does not affect antibody binding to a nearby epitope or its capacity to serve as a neutralization target [49]. Collectively, these findings suggest that ZIKV pathogenicity, but not immunogenicity, might be influenced by the glycosylation of the E protein, which occurs post-translationally in ER of the host cell. This host cell-dependent glycosylation process of ZIKV E could thus have wide implications on pathogenicity and infectivity of the virus. However, future research is required to evaluate the full extent of this circumstance. 

In regard to virus–host cell infection, in this study, we explored ZIKV interactions with cellular lectins that bind specific types of glycans we identified on the virus. DC-SIGN is a lectin-like molecule, expressed in dendritic cells and moncocytes that can facilitate virus entry through its binding affinity with the glycans on the E protein of flaviviruses [50]. Similar to most flaviviruses, N154 glycosylation was found to play a role in ZIKV infection of mammalian cells through this entry factor DC-SIGN [51,52] For DENV, DC-SIGN interacts directly with the additional N67-glycan (which is absent in other flaviviruses) on viral E protein. Indeed, the glycosaminoglycan (GAGs) of C-type lectins, including DC-SIGN, have a binding affinity as an attachment factor for host cell entry of ZIKV similar to other pathogenic flaviviruses [53]. We also observed a significant decrease in ZIKV infection in DC-SIGN- and L-SIGN-expressing cells in the presence of competitive inhibitors rich in mannose. As a result, the presence of monoclonal antibodies to various C-type lectins, the competitive inhibitors rich in mannose, or antiviral gene therapy may be able to reduce ZIKV replication by impairing the binding between viral N-glycan of E protein and GAGs of DC-SIGN. Recent studies suggest that the cells expressing DC-SIGN, such as human dermal fibroblasts, epidermal keratinocytes, and immature dendritic cells, are permissive to the most recent ZIKV isolates [54]. Taken together, our results may partially explain the alterations in the mechanisms of pathogenesis and tissue tropism of these recent ZIKV strains. Further studies are needed to determine the impact of host glycosylation variability on ZIKV pathogenesis and infectivity in vivo. 

In conclusion, our findings provide a first-of-its-kind detailed repertoire of N-glycans linked to the ZIKV envelope. To our knowledge, this is the first comprehensive mapping on the glycome of ZIKV E in different physiologically relevant cell lines. Our study shows that the N-glycans linked to ZIKV E produced by both insect and various mammalian cells are highly heterogeneous in structure and composition. Both African and Asian lineage strains showed very similar N-glycosylation patterns of ZIKV E. We also noticed that the N-glycan pattern of the ZIKV E depends on not only virus strain characteristics, but also on cell line type. We showed that ZIKV infection in DC-SIGN- and L-SIGN-expressing cells was significantly decreased in the presence of competitive inhibitors rich in mannose, suggesting an important role for these lectin-like molecules as cell entry factors in pathogenesis, potentially emerging new strains of ZIKV and alternative targets for therapeutic development. The results of this study may be important in efforts to design therapeutically active antibodies against ZIKV replication and glycomimetic compound-based antivirals.

## 4. Materials and Methods

### 4.1. Cell Lines and Viruses

The ZIKV stocks were produced in various cell lines derived from human (placenta, brain, and monocytes), monkey, and insects (Table 1), and titered in Vero (kidney epithelial cells extracted from an African green monkey) cells. ZIKV strains MR766 (Rhesus/1947/Uganda; BEI Cat. # NR-50065), PRVABC59 (Human/2015/Puerto Rico; BEI Cat. # NR-50240), FLR (Human/2015/Colombia; BEI Cat. # NR-50183), and IbH 30656 (Human/1968/Nigeria; BEI Cat. # NR-50066) were obtained from BEI Zika resources. A ZIKV Brazilian isolate (SJRP-HB-2016-1840 (KY441403.1)—herein referred to as SJRP) was also obtained from the University of Texas Medical Branch (UTMB) Arbovirus reference collection (Table 1). The cells were cultured and maintained in Dulbecco’s modified eagle medium (DMEM, Gibco, Life Technologies, Carlsbad CA, USA) supplemented with 10% heat-inactivated fetal bovine serum (FBS, Invitrogen, Carlsbad, CA, USA) at 37 °C in a 5% carbon dioxide humidified environment. The mammalian cell lines JEG-3 (human placental choriocarcinoma) and SNB-19 (human brain glioblastoma) were grown on DMEM (1X) supplemented with the 10% FBS. The LLC-MK2 (kidney epithelial cells extracted from *Macaca mulatta* monkey) cells were grown on Medium 199 (Biowest, Riverside, MO, USA) supplemented with 1% horse serum (Invitrogen, Carlsbad, CA, USA). The THP-1 (human monocytic cells) cells were grown on Roswell Park Memorial Institute (RPMI) medium (Gibco, CA, USA) supplemented with 10% FBS and 0.05 mM β-mercaptoethanol (Sigma-Aldrich, St. Louis, MO, USA). The mosquito C6/36 (*Aedes albopictus* clone) cells were grown on minimum essential medium (MEM, Gibco, Life Technologies, Carlsbad, CA, USA) supplemented with 10% FBS. All cell culture media were supplemented with 1% penicillin-streptomycin (Gibco, Carlsbad, CA, USA) and maintained at 37 °C in a 5% carbon dioxide humidified environment, except the C6/36 cells which were maintained at 28 °C.

### 4.2. Virus Infection

The various ZIKV strains, such as African strains (MR766 and IbH), Asian strains (PRVABC59 and FLR), and a primary isolate from Brazil in 2016 (SJRP), at multiplicity of infection (MOI) of 1 were adsorbed onto cells. After 90 min, in serum-free medium with rocking every 15 min, the inoculum was removed; the C6/36, Vero, LLC-MK2, SNB-19, THP-1, and JEG-3 cells were maintained in respective medium containing 2% FBS. All the cells lines were maintained at 37 °C in a CO_2_ incubator (except the C6/36 cells, which were maintained at 28 °C). For large-scale virus production, all cells were maintained at 37 °C in a CO_2_ incubator for 3–4 days (except C6/36, cells which were maintained at 28 °C for 5–7 days) until cytopathic effects were observed.

### 4.3. Purification of ZIKV Virus and Isolation of E Protein

A large stock of ZIKV viruses were purified using sucrose cushion in ultracentrifugation as described in previous methods [55]. Briefly, culture supernatants were centrifuged at 6000 rpm for 10 min, and a 0.45 μm filter was used to remove cell debris. Virus-containing filtrate was layered onto 20% (w/v) sucrose and subjected to ultracentrifugation at 100,715× *g*, 4 °C for 3.5 h. Pellets were dissolved in 100 μL of NTE buffer (10 mM Tris-HCl, pH 8.0, 120 mM NaCl, and 1 mM EDTA (ethylene diamine tetra acetic acid)). The purified virions were then resolved on gradient 4–12% bis-tris protein gels. The presence of ZIKV glycoprotein E was confirmed by Western blot analysis using rabbit polyclonal antibody (1:1000) against the E protein of ZIKV (Genetex Inc., Irvine, CA, USA), and confirmed band was excised for MS analysis. 

### 4.4. Lectin Microarray

For the lectin array analysis, cells (C6/36 cells, Vero cells, SNB-19 cells, and JEG-3 cells) grown until they reached 60–70% confluency were incubated with various ZIKV strains, such as African strains (MR766 and IbH), Asian strains (PRVABC59 and FLR), and the primary Brazilian isolate SJRP, at MOI of 1 in serum-free medium at 37 °C for 90 min, and control uninfected (no virus added) cells were maintained under similar conditions. After 90 min, the inoculum was removed and cells continued to culture in their respective culture conditions. After the infection (similar infection conditions were followed), the uninfected cells and ZIKV-infected cells, separately, were harvested using cell scrapper, made into a single cell suspension, and counted using cell countess (Invitrogen, Carlsbad, CA, USA). The lectin array was performed on ZIKV-infected cells, and also on uninfected control cells. The lectin microarray enabled sensitive analysis of multiple glycan structures (45 structures) by employing a panel of immobilized lectins with known glycan binding specificity [56,57,58,59,60,61,62,63,64]. Cell surface proteins were purified using Mem-PER™ Plus Membrane Protein Extraction Kit (Thermo Fisher Scientific, Rockford, lL, USA). Isolated proteins were labeled with Cy3 dye and hybridized to a lectin microarray [56,57,58,59,60,61,62,63,64]. The resulting lectin chips were scanned for fluorescence intensity on each lectin-coated spot using an evanescent-field fluorescence scanner. Data were normalized using the global normalization method.

### 4.5. Mass Spectrometry Identification of N-linked Glycans and N-glycan Preparation

Gel bands stained with Coomassie Blue were excised and transferred into clean tubes. Then, 200 µL of 50 mM ammonium bicarbonate (AMBIC) (Sigma-Aldrich, St Louis, MO, USA) and 200 µL of acetonitrile (Sigma-Aldrich, St. Louis, MO, USA) were added to the samples, mixed thoroughly, and incubated at room temperature (RT) for 5 min. The supernatants were then discarded, and the washing step was repeated once more. The gel pieces were dried with a vacuum centrifuge for 10 min. Then, 200 µL of a 10 mM DTT (1,4-Dithiothreitol, Sigma-Aldrich, St. Louis, MO, USA) solution was added and incubated at 50 °C for 30 min. The DTT solution was discarded, and the samples were briefly washed with 200 µL of acetonitrile solution. Again, the samples were dried with a vacuum centrifuge for 10 min. 

After drying, the samples were incubated with 200 µL of a 55 mM IAA (Iodoacetamide, Sigma-Aldrich, St. Louis, MO, USA) solution for 30 min in the dark at RT. The IAA solution was then discarded, and the samples were washed with 500 μL of 50 mM AMBIC for 15 min at RT, followed by 5 min incubation with 200 μL of acetonitrile. The samples were dried once again with a vacuum centrifuge for 10 min, before adding 500 μL of 50 mM AMBIC containing 10 μg of TPCK-treated trypsin (Sigma-Aldrich, St. Louis, MO, USA). After incubating overnight at 37 °C, the trypsin digestion was terminated by boiling the sample for 3 min.

The supernatants were recovered and collected in a clean glass tube, before carrying out two sequential washes with 200 μL of 50 mM AMBIC, vortexed for 15 min; 200 μL of 50% acetonitrile in 50 mM AMBIC, vortexed for 15 min; and 200 μL of acetonitrile, vortexed for 15 min. For each sample, all washes were collected, pooled in the same glass tube that was previously used, and then lyophilized. The dried materials were resuspended in 200 μL of 50 mM AMBIC. Then, 1 μL of PNGaseF (New England Biolabs, Ipswich, MA, USA) was added to this for an overnight incubation at 37 °C. Two drops of 5% acetic acid (Fisherbrand, Waltham, MA, USA) were added to stop the enzymatic reaction before purifying the released N-glycan over a C18 Sep-Pak (50 mg) column (Waters, Milford, MA, USA) that was conditioned with 1 column volume (CV) of methanol (Sigma-Aldrich, St. Louis, MO, USA), 1 CV of 5% acetic acid, 1 CV of 1-propanol (Sigma-Aldrich), and 1 CV of 5% acetic acid. The C18 column was washed with 3 mL of 5% acetic acid, flow through; and wash fractions were collected, pooled, and lyophilized. 

#### 4.5.1. Permethylation of N-glycan

Lyophilized N-glycan samples were incubated with 1 mL of a DMSO (Dimethyl Sulfoxide; Sigma)–NaOH (Sigma-Aldrich, St. Louis, MO, USA) slurry solution and 500 µl of methyl iodide (Sigma-Aldrich, St. Louis, MO, USA) for 20–30 min under vigorous shaking at RT. Then, 1 mL of Milli-Q water was added to stop the reaction, and 1 mL of chloroform (Sigma-Aldrich, St. Louis, MO, USA) was added to purify the permethylated N-glycan. Next, 3 mL of Milli-Q water was added to wash the chloroform fractions, and the mixture was briefly vortexed. The water was discarded by additional centrifugation. This wash step was repeated 3 times. The chloroform fraction was dried before being redissolved in 200 mL of 50% methanol. This was then loaded into a conditioned (1 CV methanol, 1 CV Milli-Q water, 1 CV acetonitrile (Sigma-Aldrich, St. Louis, MO, USA), and 1 CV Milli-Q Water) C18 Sep-Pak (200 mg) column. The C18 column was washed with 6 mL of 15% acetonitrile and then eluted with 6 mL of 50% acetonitrile. The eluted fraction was lyophilized and then redissolved in 10 µl of 75% methanol, from which 1 µl was mixed with 1 μL DHB (2,5-dihydroxybenzoic acid (Sigma-Aldrich, St. Louis, MO, USA)) (5mg/mL in 50% acetonitrile with 0.1% trifluoroacetic acid (Sigma-Aldrich, St. Louis, MO, USA)) and spotted on a MALDI polished steel target plate (Bruker Daltonics, Bremen, Germany).

#### 4.5.2. MS Data Acquisition and Analyses

MS data were acquired on a Bruker UltraFlex II MALDI-TOF Mass Spectrometer instrument. The reflective positive mode was used, and data were recorded between 500 and 6000 *m/z*. For each MS N-glycan profile, the aggregation of 20,000 laser shots or more were considered for data extraction. Mass signals of a signal/noise ratio of at least four were considered, and only MS signals matching an N-glycan composition were considered for further analysis. Subsequent MS post-data acquisition analysis was made using mMass [65]. The relative abundance of each N-glycans identified on ZIKV E in each experimental condition was calculated based on the absolute intensity of the first isotopic peak of a given N-glycan relative to the sum of all N-glycan intensities.

#### 4.5.3. Assays to Evaluate the Functional Importance of N-linked Glycans

In inhibition experiments, 3T3, 3T3-DCSIGN, and 3T3-L-SIGN cells were incubated with 200 μg/mL of yeast mannan (Sigma-Aldrich, St. Louis, MO, USA) or 0.5 mM EDTA at 37 °C for 30 min. Various strains of Zika virus (MOI = 1) produced from C6/36 cell line were pre-incubated with the same concentration of mannan inhibitor and adsorbed on pretreated cells. The mannan treatment was maintained constant throughout the duration of infection, whereas the EDTA and virus-containing medium were removed after 2 h and the infection was continued with the fresh medium. The infected cells were detected using viral E protein antibody immunofluorescence assay and the percent of infected cells was quantified by using an Operetta High-Content Imaging System (PerkinElmer, Inc., Waltham, MA, USA).

The Immunofluorescence assay was performed as follows. The C6/36, Vero, and SNB-19 cells were grown on cover slips and mock-infected or infected with various strains of ZIKV. After 24 h post-infection, cells were processed for indirect immunofluorescence assay using the double-labeling method. Briefly, cells were fixed in 4% paraformaldehyde and processed for immunofluorescence assay. The cells were blocked and permeabilized using 5% goat serum (Sigma-Aldrich, St. Louis, MO, USA) with 0.5% Triton X-100 (Sigma-Aldrich, St. Louis, MO, USA). The cells were then immunoassayed using rabbit polyclonal antibody (1:1000) against the E protein of ZIKV (Genetex Inc., Irvine, CA, USA) in a combination with ER, using ER ID dye as per the manufacturer’s instructions (Enzo Life Sciences, Inc, Farmingdale, NY, USA), and the cell nuclei (1:2000), using Hoechst stain (Invitrogen, Rockford, IL, USA) for 15 min. Subsequently, Alexa Fluor 594-conjugated secondary antibody (1:2000) (Invitrogen, Rockford, IL, USA) was added onto cells and images were captured under 40× using the Operetta High-Content Imaging System (PerkinElmer, Waltham, MA, USA). In between each step, the cells were washed with 1× PBS two times for 5 min.

#### 4.5.4. Statistical Analysis

Spearman’s *r* Rank Order Correlations were conducted using GraphPad Prism release 7.0 (GraphPad Software, San Diego, CA, USA).

## Figures and Tables

**Table 1:** A list of cell lines and viruses used for this study and their origin is represented in table format. Various cells lines (upper part of the table) and Zika virus strains (lower part of the table) used in this study to identify strain-specific N-linked glycans of glycoprotein (E) of ZIKV.

Cell Types and Details			
**Cell Type**	Vero Cells	THP-1 Cells	C6/36 Cells
**Organism**	Cercopithecus Aethiops	Homo Sapiens, Human	Aedes Albopictus, Mosquito
**Tissue**	Kidney	Peripheral Blood	Larva, Whole
**Morphology**	Epithelial	Monocyte	Larva
**Culture Properties**	Adherent	Suspension	Adherent
**Cell Type**	LLC-MK2 Cells	SNB-19 Cells	JEG-3 Cells
**Organism**	Macaca Mulatta, Monkey, Rhesus	Homo Sapiens	Homo Sapiens
**Tissue**	Kidney	Brain	Placenta
**Morphology**	Epithelial	Astrocytoma/Fibroblastic	Epithelial
**Culture Properties**	Adherent	Adherent	Adherent
**Viral Strains and Details**			
**Zika Virus Strain**	Origin	Genbank ID	Lineage
**Mr766**	Human, Uganda, 1947	Ku963573.2	African
**Flr**	Human, Colombia, 2015	Kx087102.2	Asian
**Prvabc59**	Human, Puerto Rica, 2015	Kx087101.3	Asian
**Ibh**	Human/1968/Nigeria	KU963574.2	African
**Sjrp-Hb-2016-1840**	Primary Isolate	Ky441403.1	Brazilian

## Supplementary Materials

Supplementary materials can be found at https://www.mdpi.com/1422-0067/20/20/5206/s1: Figure S1. SDS-PAGE and western blotting analysis of purified Zika virions; Table S1. N-glycans of envelope (E) protein of matures ZIKV identified by MALDI-TOF; Table S2. N-glycans of envelope (E) protein of mature ZIKV identified by lectin microarray; Table S3. Lectins used for 45 lectin microarray, and their names and glycan binding specificities.
