# Supplementary material for: Glycosylation of Zika Virus is Important in Host–Virus Interaction and Pathogenic Potential
Source: Int J Mol Sci. 2019 Oct 21;20(20):5206. doi: 10.3390/ijms20205206 (PMC6829355; doi:10.3390/ijms20205206)
Supplement: Supplementary file 1 [file ijms-20-05206-s001.pdf]

Supplementary Information

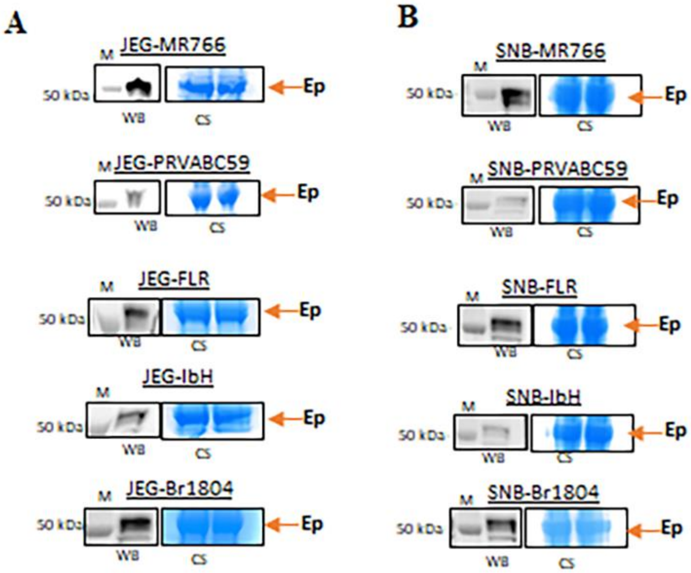

Figure S1. SDS-PAGE and Western blotting analysis of purified Zika virions. Asian strains (PRVABC59 and FLR), African strains (MR766 and IbH), and the primary isolate (SJRP-HB-2016-1840) produced from (**A**) JEG-3 cells and (**B**) SNB-19 cells and stained using mass spectrometry compatible Coomassie (CS) dye after resolving on SDS-PAGE. The Zika virus envelope proteins (Ep) were excised from the band for N-linked glycan analysis. Western blotting (WB) was performed to confirm the envelope proteins of ZIKV virions using Zika virus envelope protein antibody.

Table S1. N-glycans of envelope (E) protein of mature ZIKV identified by MALDI-ToF. Predicted N-linked glycan structures and their molecular ions in MALDI spectra of envelope (E) protein obtained from purified mature ZIKV virions produced from cell lines of different origin.

Mass (m/z)		Composition	Proposed Structure
Th.	Obs.		
1579.9	1579.9	Hex5HexNAc2	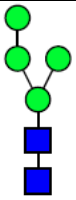
1620.9	1620.9	Hex4HexNAc3	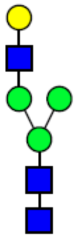
1661.9	1662.0	Hex3HexNAc4	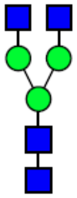
1784.0	1784.0	Hex6HexNAc2	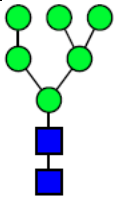
1825.0	1825.0	Hex5HexNAc3	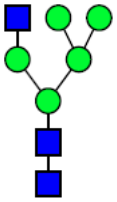
1836.0	1836.1	Hex3HexNAc4Fuc1	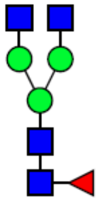
1866.1	1866.1	Hex4HexNAc4	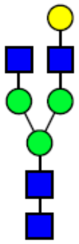

1907.1	1907.1	Hex3HexNAc5	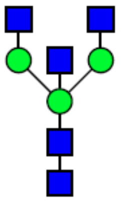
1988.1	1988.1	Hex7HexNAc2	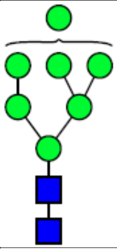
1999.1	1999.1	Hex5HexNAc3Fuc1	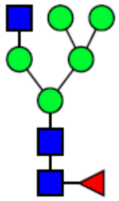
2029.1	2029.1	Hex6HexNAc3	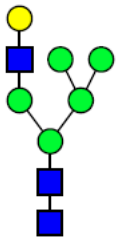
2040.2	2040.2	Hex4HexNAc4Fuc1	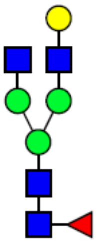
2070.2	2070.2	Hex5HexNAc4	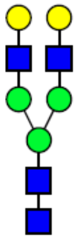
2192.2	2192.2	Hex8HexNAc2	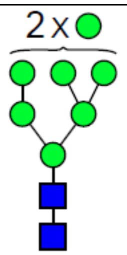

2244.3	2244.3	Hex5HexNAc4Fuc1	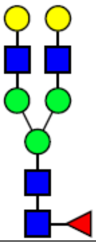
2285.3	2285.3	Hex4HexNAc5Fuc1	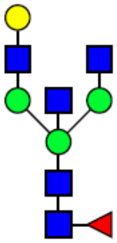
2390.3	2390.3	Hex6HexNAc3NeuAc1	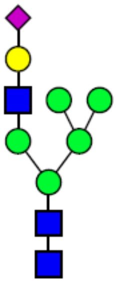
2396.3	2396.3	Hex9HexNAc2	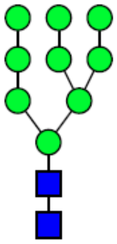
2401.4	2401.4	Hex4HexNAc4Fuc1NeuAc1	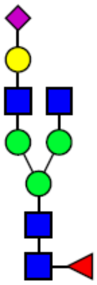
2431.4	2431.4	Hex5HexNAc4NeuAc1	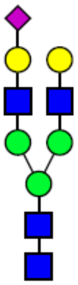

2461.4	2461.4	Hex5HexNAc4NeuGc1	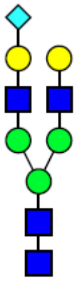
2489.4	2489.4	Hex5HexNAc5Fuc1	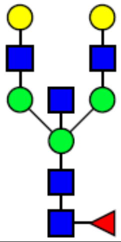
2519.4	2519.4	Hex6HexNAc5	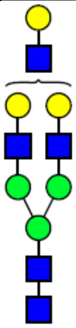
2605.5	2605.5	Hex5HexNAc4Fuc1NeuAc1	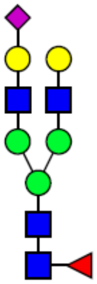
2635.5	2635.5	Hex6HexNAc4NeuAc1	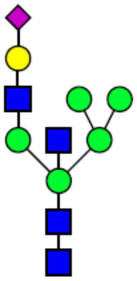

2652.5	2652.5	Hex7HexNAc4Fuc1	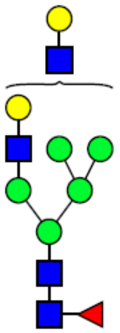
2676.5	2676.5	Hex5HexNAc5NeuAc1	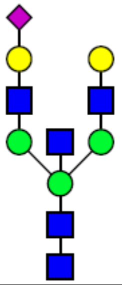
2693.5	2693.5	Hex6HexNAc5Fuc1	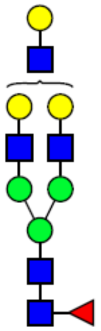
2738.5	2738.5	Hex6HexNAc3Fuc2NeuAc1	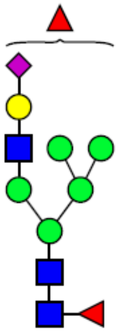
2792.6	2792.5	Hex5HexNAc4NeuAc2	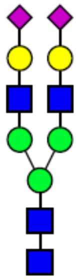

2880.6	2880.6	Hex6HexNAc5NeuAc1	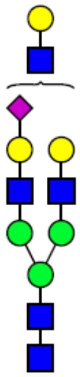
2966.7	2966.7	Hex5HexNAc4Fuc1NeuAc2	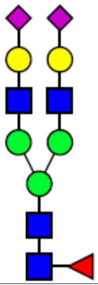
2996.7	2996.7	Hex6HexNAc4NeuAc2	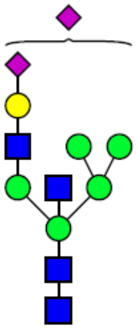
3037.7	3037.7	Hex5HexNAc5NeuAc2	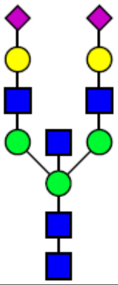
3054.7	3054.7	Hex6HexNAc5Fuc1NeuAc1	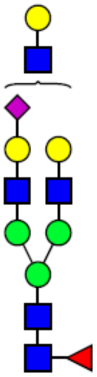

3112.8	3112.8	Hex6HexNAc6Fuc2	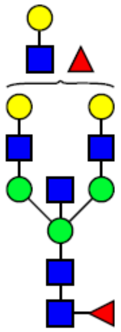
3153.8	3153.8	Hex5HexNAc4NeuAc3	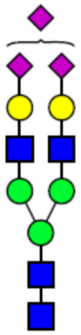
3187.8	3187.8	Hex7HexNAc4Fuc2NeuAc1	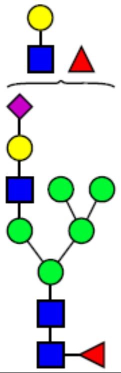
3241.8	3241.8	Hex6HexNAc5NeuAc2	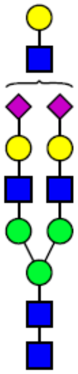

3357.9	3357.9	Hex6HexNAc7Fuc2	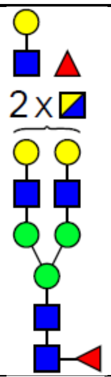
3415.9	3415.9	Hex6HexNAc5Fuc1NeuAc2	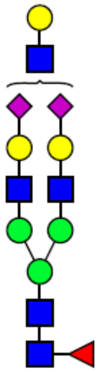
3504.0	3504.0	Hex7HexNAc6Fuc1NeuAc1	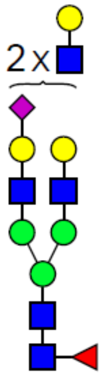
3549.0	3549.1	Hex7HexNAc4Fuc2NeuAc2	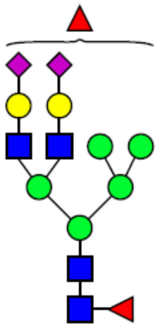

3603.0	3603.0	Hex6HexNAc5NeuAc3	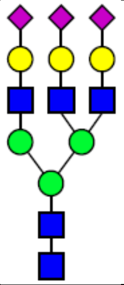
3777.1	3777.1	Hex6HexNAc5Fuc1NeuAc3	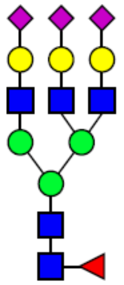
3865.2	3865.1	Hex7HexNAc6Fuc1NeuAc2	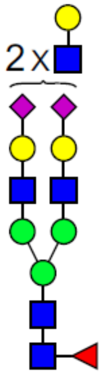
3964.2	3964.2	Hex6HexNAc5NeuAc4	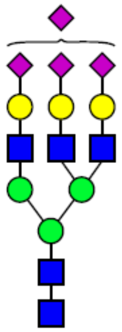
4052.3	4052.2	Hex7HexNAc6NeuAc3	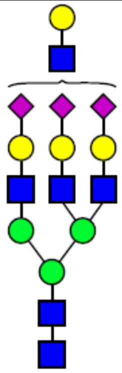

4138.3	4138.3	Hex6HexNAc5Fuc1NeuAc4	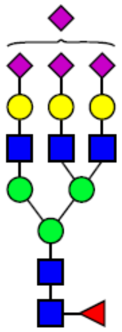
4226.4	4226.4	Hex7HexNAc6Fuc1NeuAc3	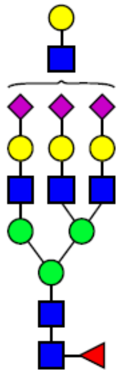
4325.4	4325.4	Hex6HexNAc5NeuAc5	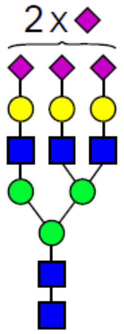
4413.5	4413.5	Hex7HexNAc6NeuAc4	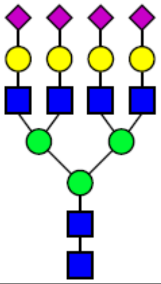

Table S2. N-glycans of envelope (E) protein of mature ZIKV identified by lectin microarray. Predicted N-linked glycan molecular ions of envelope (E) protein obtained from purified mature ZIKV virions produced from cell lines of different origin based on their binding specificities in lectin microarray.

m/z (ZVEG-E)	Lectin (cell surface)	Spearman r	*p*-Value	N
1620.9	UEA_I	–0.615*	0.025	13
	ECA	–0.698**	0.008	13
	TxLC_I	–0.578*	0.039	13
	PWM	–0.698**	0.008	13

	ACA	0.687**	0.010	13
	MPA	0.593*	0.033	13
1662.0	SNA	–0.648*	0.043	10
	GNA	0.648*	0.043	10
	ACG	–0.661*	0.038	10
	ACA	0.697*	0.025	10
1784.0	AOL	–0.560*	0.046	13
1825.0	UEA_I	–0.608*	0.036	12
	AOL	–0.727**	0.007	12
	AAL	–0.678*	0.015	12
	MAL_I	–0.636*	0.026	12
	TxLC_I	–0.581*	0.047	12
	BPL	0.608*	0.036	12
	ACA	0.678*	0.015	12
	MPA	0.657*	0.020	12
1836.1	MAL_I	–0.648*	0.043	10
	SNA	–0.758*	0.011	10
	PHAL	–0.851**	0.002	10
	ConA	0.842**	0.002	10
	GNA	0.770**	0.009	10
	HHL	0.733*	0.016	10
	TJA_II	0.673*	0.033	10
	UDA	0.879**	0.001	10
1907.1	TJA_II	0.685*	0.014	12
	PNA	0.699*	0.011	12
1999.1	PSA	0.650*	0.042	10
	LCA	0.663*	0.037	10
	MAL_I	–0.657*	0.039	10
	ACG	–0.748*	0.013	10
	ACA	0.705*	0.023	10
	SBA	0.671*	0.034	10
	Calsepa	0.754*	0.012	10
	WGA	–0.675*	0.032	10
2029.155244	AOL	–0.741**	0.006	12
	AAL	–0.678*	0.015	12
	PNA	0.615*	0.033	12
	ACA	0.615*	0.033	12
	MPA	0.622*	0.031	12
2070.184523	DSA	0.488*	0.034	19
	PWM	–0.488*	0.034	19
	VVA	–0.474*	0.041	19
2192.2	AOL	–0.599*	0.031	13
2244.282958	DSA	0.486*	0.030	20
	VVA	–0.553*	0.011	20
	WGA	0.490*	0.028	20
2285.3	MAL_I	–0.618*	0.043	11

2431.372544	PSA	–0.575*	0.013	18
	LCA	–0.505*	0.033	18
	AOL	0.488*	0.040	18
	AAL	0.494*	0.037	18
	MAL_I	0.647**	0.004	18
	SNA	0.649**	0.004	18
	SSA	0.560*	0.016	18
	PHAL	0.508*	0.031	18
	RCA120	0.606**	0.008	18
	PHAE	0.655**	0.003	18
	DSA	0.779**	0.000	18
	NPA	–0.487*	0.040	18
	ConA	–0.573*	0.013	18
	GNA	–0.759**	0.000	18
	HHL	–0.767**	0.000	18
	BPL	–0.511*	0.030	18
	TJA_II	–0.612**	0.007	18
	ABA	–0.571*	0.013	18
	UDA	–0.589*	0.010	18
	PNA	–0.587*	0.010	18
	ACA	–0.641**	0.004	18
	MPA	–0.505*	0.033	18
	VVA	–0.630**	0.005	18
	Calsepa	–0.496*	0.036	18
	WGA	0.647**	0.004	18
	GSL_I_A4	–0.517*	0.028	18
2605.468092	PSA	–0.718**	0.002	16
	LCA	–0.618*	0.011	16
	UEA_I	0.789**	0.000	16
	AOL	0.738**	0.001	16
	AAL	0.721**	0.002	16
	MAL_I	0.638**	0.008	16
	ECA	0.550*	0.027	16
	PHAE	0.588*	0.017	16
	DSA	0.571*	0.021	16
	GSL_II	0.557*	0.025	16
	NPA	–0.628**	0.009	16
	GNA	–0.512*	0.043	16
	HHL	–0.509*	0.044	16
	ACG	0.529*	0.035	16
	TxLC_I	0.698**	0.003	16
	BPL	–0.653**	0.006	16
	TJA_II	–0.506*	0.046	16
	ABA	–0.894**	0.000	16
	Jacalin	–0.653**	0.006	16
	PNA	–0.568*	0.022	16

	WFA	0.690**	0.003	16
	ACA	–0.897**	0.000	16
	MPA	–0.853**	0.000	16
	VVA	–0.604*	0.013	16
	Calsepa	–0.741**	0.001	16
	MAH	0.738**	0.001	16
	WGA	0.800**	0.000	16
	GSL_I_A4	–0.510*	0.043	16
2635.482067	PHAL	0.589*	0.034	13
	RCA120	0.553*	0.050	13
	NPA	–0.572*	0.041	13
2693.522874	PSA	–0.842**	0.002	10
	UEA_I	0.721*	0.019	10
	AOL	0.661*	0.038	10
	TxLC_I	0.815**	0.004	10
	BPL	–0.685*	0.029	10
	EEL	0.646*	0.043	10
	ABA	–0.794**	0.006	10
	PWM	0.927**	0.000	10
	ACA	–0.733*	0.016	10
	MPA	–0.806**	0.005	10
	MAH	0.640*	0.046	10
	WGA	0.721*	0.019	10
2966.7	UEA_I	0.852**	0.001	11
2996.674682	AOL	0.836**	0.001	11
	AAL	0.827**	0.002	11
	TxLC_I	0.793**	0.004	11
	BPL	–0.673*	0.023	11
	ABA	–0.818**	0.002	11
	PWM	0.682*	0.021	11
	WFA	0.825**	0.002	11
	ACA	–0.682*	0.021	11
	MPA	–0.818**	0.002	11
	MAH	0.703*	0.016	11
	WGA	0.673*	0.023	11
	STL	–0.770*	0.015	9
3037.687563	LTL	0.557*	0.031	15
	AOL	–0.661**	0.007	15
	AAL	–0.693**	0.004	15
	MAL_I	–0.557*	0.031	15
	DSA	–0.614*	0.015	15
	GNA	0.543*	0.037	15
	HHL	0.550*	0.034	15
	BPL	0.671**	0.006	15
	TJA_II	0.607*	0.016	15
	PNA	0.554*	0.032	15

	ACA	0.543*	0.037	15
	PTL_I	0.517*	0.048	15
	WGA	–0.671**	0.006	15
3054.708381	UEA_I	0.768**	0.001	14
	AOL	0.701**	0.005	14
	AAL	0.679**	0.008	14
	TxLC_I	0.755**	0.002	14
	BPL	–0.657*	0.011	14
	ABA	–0.675**	0.008	14
	ACA	–0.688**	0.007	14
	MPA	–0.749**	0.002	14
	MAH	0.652*	0.012	14
	WGA	0.631*	0.016	14
3357.870189	TJA_I	–0.723*	0.018	10
	NPA	0.784**	0.007	10
	ACG	–0.675*	0.032	10
	HPA	0.685*	0.029	10
	VVA	0.793**	0.006	10
3415.91826	UEA_I	0.626*	0.022	13
	AOL	0.736**	0.004	13
	AAL	0.742**	0.004	13
	BPL	–0.758**	0.003	13
	ABA	–0.621*	0.024	13
	LEL	0.582*	0.037	13
	ACA	–0.610*	0.027	13
	MPA	–0.643*	0.018	13
	WGA	0.621*	0.024	13
3603.00794	DSA	–0.502*	0.029	19
	EEL	0.456*	0.050	19
	VVA	0.577**	0.010	19
3777.1	LTL	0.619*	0.024	13
	UEA_I	0.738**	0.004	13
	AOL	0.726**	0.005	13
	AAL	0.685**	0.010	13
	TxLC_I	0.680*	0.011	13
	BPL	–0.630*	0.021	13
	ABA	–0.669*	0.012	13
	LEL	0.580*	0.038	13
	PWM	0.710**	0.007	13
	MPA	–0.710**	0.007	13
	MAH	0.629*	0.021	13
3865.144605	ECA	–0.900*	0.037	5
	STL	0.975**	0.005	5
	ACA	0.900*	0.037	5
3964.2	MAL_I	–0.574*	0.016	17
	SNA	–0.529*	0.029	17

	RCA120	–0.493*	0.045	17
	PHAE	–0.515*	0.035	17
	DSA	–0.733**	0.001	17
	ConA	0.615**	0.009	17
	GNA	0.708**	0.001	17
	HHL	0.725**	0.001	17
	BPL	0.505*	0.039	17
	TJA_II	0.625**	0.007	17
	PNA	0.588*	0.013	17
	ACA	0.596*	0.012	17
	VVA	0.620**	0.008	17
	WGA	–0.527*	0.030	17
4052.3	UEA_I	0.546*	0.029	16
	AOL	0.659**	0.005	16
	AAL	0.558*	0.025	16
	MPA	–0.508*	0.045	16
4325.5	SNA	0.900*	0.037	5
4413.518836	DSA	–0.515*	0.050	15
	HHL	0.520*	0.047	15

Table S3. Lectins used for 45 lectin microarray, and their names and glycan binding specificities. A list of lectins, binding specificities, origin, and abbreviations of 45 lectins on the Lectin chip examined in this study is shown. Abbreviations: Gal (D-galactose), GalNAc (N-acetyl-galactosamine), GlcNAc (N-acetyl-glucosamine), Fuc (L-fucose), Glc (D-glucose), Sia (Sialic acid), LacNAc (N-acetyl-lactosamine). Specificity data were obtained by frontal affinity chromatography and glycoconjugate microarray.

	Name	Species	Origin	Glycan specificity [2]
1	LTL	*Lotus tetragonolobus*	Natural	Fuc (Le^x, Le^y)
2	PSA	*Pisum sativum*	Natural	α1-6Fuc up to biantenna
3	LCA	*Lens culinaris*	Natural	α1-6Fuc up to biantenna
4	UEAI	*Ulex europaeus*	Natural	α1-2Fuc
5	AOL	*Aspergillus oryzae*	Recombinant	α1-6Fuc (Core), α1-2Fuc (H), α1-3Fuc (Le^x), α1-3Fuc (Le^a)
6	AAL	*Aleuria aurantia*	Natural	α1-6Fuc (Core), α1-2Fuc (H), α1-3Fuc (Le^x), α1-3Fuc (Le^a)
7	MAL	*Maackia amurensis*	Natural	α2-3Sia
8	SNA	*Sambucus nigra*	Natural	α2-6Sia
9	SSA	*Sambucus sieboldiana*	Natural	α2-6Sia
10	TJAI	*Trichosanthes japonica*	Natural	α2-6Sia
11	PHAL	*Phaseolus vulgaris*	Natural	GlcNAcβ1-6Man (Tetraantenna)
12	ECA	*Erythrina cristagalli*	Natural	βGal
13	RCA120	*Ricinus communis*	Natural	βGal
14	PHAE	*Phaseolus vulgaris*	Natural	bisecting GlcNAc
15	DSA	*Datura stramonium*	Natural	GlcNAcβ1-6Man (Tetraantenna)
16	GSLII	*Griffonia simplicifolia*	Natural	GlcNAcβ1-4Man

17	NPA	*Narcissus pseudonarcissus*	Natural	Manα1-3Man
18	ConA	*Canavalia ensiformis*	Natural	M3, Manα1-2Manα1-3(Manα1-6)Man, GlcNAcβ1-2Manα1-3(Manα1-6)Man
19	GNA	*Galanthus nivalis*	Natural	Manα1-3Man, Manα1-6Man
20	HHL	*Hippeastrum hybrid*	Natural	Manα1-3Man, Manα1-6Man
21	ACG	*Agrocybe cylindracea*	Natural	α2-3Sia
22	TxLcI	*Tulipa gesneriana*	Natural	Galactosylated N-glycans up to triantenna
23	BPL	*Bauhinia purpurea alba*	Natural	Galβ1-3GlcNAc(GalNAc), α/βGalNAc
24	TJAII	*Trichosanthes japonica*	Natural	α1-2Fuc
25	EEL	*Euonymus europaeus*	Natural	αGal (B)
26	ABA	*Agarics bisporus*	Natural	Galβ1-3GalNAc (T), GlcNAc
27	LEL	*Lycopersicon esculentum*	Natural	Polylactosamine, (GlcNAc)n
28	STL	*Solanum tuberosum*	Natural	Polylactosamine, (GlcNAc)n
29	UDA	*Urtica dioica*	Natural	(GlcNAc)n
30	PWM	*Phytolacca americana*	Natural	(GlcNAc)n
31	Jacalin	*Artocarpus integrifolia*	Natural	Galβ1-3GalNAc (T), GalNAcα (Tn)
32	PNA	*Arachis hypogaea*	Natural	Galβ1-3GalNAc (T)
33	WFA	*Wisteria floribunda*	Natural	Terminal GalNAc, LacDiNAc
34	ACA	*Amaranthus caudatus*	Natural	Galβ1-3GalNAc (T)
35	MPA	*Maclura pomifera*	Natural	Galβ1-3GalNAc (T), GalNAca (Tn)
36	HPA	*Helix pomatia*	Natural	αGalNAc (A, Tn)
37	VVA	*Vicia villosa*	Natural	α,βGalNAc (A, Tn, LacDiNAc)
38	DBA	*Dolichos biflorus*	Natural	α,βGalNAc (A, Tn, LacDiNAc)
39	SBA	*Glycine max*	Natural	α,βGalNAc (A, Tn, LacDiNAc)
40	Calsepa	*Calystegia sepium*	Natural	Biantenna with bisecting GlcNAc
41	PTL I	*Psophocarpus tetragonolobus*	Natural	αGalNAc (A, Tn)
42	MAH	*Maackia amurensis*	Natural	α2-3Sia
43	WGA	*Triticum vulgaris*	Natural	(GlcNAc)n, polySia
44	GSLIA4	*Griffonia simplicifolia*	Natural	αGalNAc (A, Tn)
45	GSLIB4	*Griffonia simplicifolia*	Natural	αGal (B)

Abbreviations: Gal (D-galactose), GalNAc (N-acetyl-galactosamine), GlcNAc (N-acetyl-glucosamine), Fuc (L-fucose), Glc (D-glucose), Sia (Sialic acid), LacNAc (N-acetyl-lactosamine). [2] Specificity data were obtained by frontal affinity chromatography and glycoconjugate microarray.
